# Supplementary material for: Mapping of Human Polyomavirus in Renal Cell Carcinoma Tissues
Source: Int J Mol Sci. 2024 Jul 27;25(15):8213. doi: 10.3390/ijms25158213 (PMC11312419; doi:10.3390/ijms25158213)
Supplement: Supplementary file 1 [file ijms-25-08213-s001.zip › ijms-3087234-supplementary.pdf]

Supplementary Table:

Table S1: Summary and list of epitope binding site of known human polyomaviruses with antibody of CM2B4 (A) and Pab416 (B).

	HPyVs	Epitope (LT-Ag) binding region	GenBank
CM2B4 (A)	MCPyV	SRSRKPSSNASRGA	EU375803.1
	HPyV6	EHLSASEEEDNVDP	HM011560
	HPyV7	ELSSSDEEEPAASA	HM011566
	BKV	EDMFASDEEATADS	NC_001538.1
	JCV	EEMFASDDENTGSQ	NC_001699.1
	TSPyV	QDPDLFCHESTIPS	NC_014361.1
PAb416 (B)	HPyVs	Epitope (LT-Ag) binding region	GenBank
	SV40	WEQWW	NC_001669
	HPyV6	WEQWW	HM011560
	QPyV	WDKWW	BK010702
	HPyV7	WDQWW	HM011566
	WUPyV	WDYWW	NC_009539
	STLPyV	WEQWW	KF525270
	HPyV10	WDQWW	JX262162
	KIPyV	WDEWW	NC_009238
	BKV	WESWW	NC_001538.1
	JCV	WESWW	NC_001699.1
	TSPyV	WASWW	NC_014361.1
	HPYV9	WGKWW	NC_015150
	MCPyV	FKEWW	EU375803.1

Supplementary Figures:

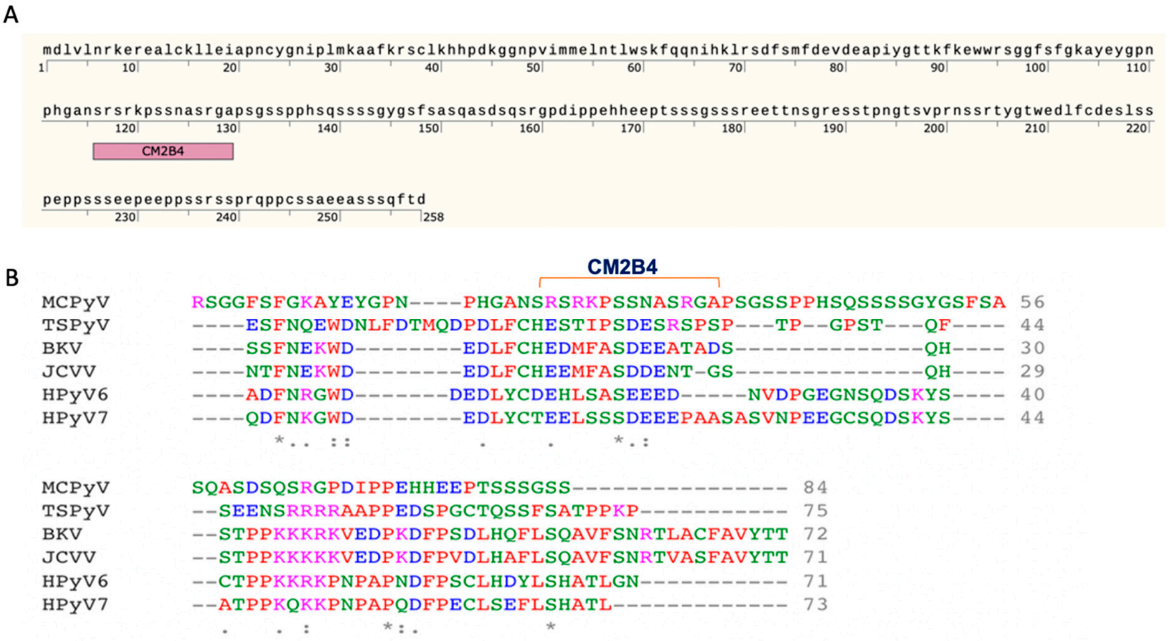

Figure S1: (A) amino acid Large T-Ag of MCPyV (MCC350), GenBank:EU375803.1 (SnapGene.com). **(B)**amino acid alignment of known human polyomaviruses. MCPyV region containing epitope site of CM2B4 is not conserved with these other polyomaviruses (Clustal multiple sequence alignment).

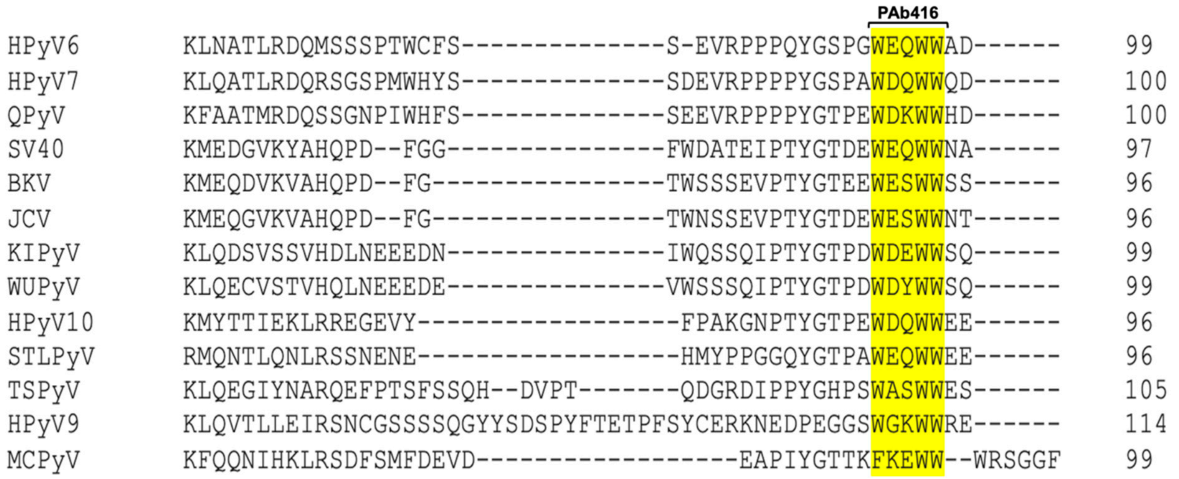

Figure S2: Amino acid alignment of Large T-Ag of some known human polyomaviruses. Region containing epitope site of PAb416 is conserved with these human polyomaviruses except MCPyV. (Clustal multiple sequence alignment)
